# Adjacent segment degeneration after superior facet joint violation of the lumbar spine

**DOI:** 10.1016/j.xnsj.2025.100843

**Published:** 2025-12-22

**Authors:** Conor McNamee, Bryan Magee, Richard N. Storey, Jake M. McDonnell, Stacey Darwish, Joseph S. Butler

**Affiliations:** a National Spine Injuries Unit, Mater Misericordiae University Hospital, Dublin 7, Ireland; b University College Dublin, Dublin 4, Ireland; c Saint Vincent’s University Hospital, Dublin 4, Ireland

**Keywords:** Facet joint violation, FJV, Pedicle screw, Lumbar fusion, Adjacent segment disease, ASD

## Abstract

**Background:**

Facet joint violation (FJV) is a recognized complication of pedicle screw fixation and has been proposed as a driver of adjacent segment disease (ASD). Biomechanical models suggest that FJV may alter segmental kinematics, but its clinical impact on degeneration and reoperation remains uncertain. This work evaluates whether superior FJV is associated with an increased risk of radiographic ASD or reoperation after lumbar fusion.

**Methods:**

Retrospective cohort study of patients undergoing lumbar pedicle screw fixation with postoperative CT imaging that captured the instrumentation and the cranial adjacent segment. Superior FJV was graded using an established 3-tier system. Follow-up imaging was assessed for disc height loss, progression of spondylolisthesis, coronal deformity, central stenosis, lateral recess height and a composite degeneration endpoint; reoperations were recorded. Propensity score weighting balanced measured covariates, and a weighted cox regression was used for time-to-event analyses.

**Results:**

Seventy-one patients met inclusion criteria, with FJV identified in 35 (49.3%). Weighted analyses demonstrated no significant association between FJV and disc height loss (HR 1.21, 95% CI 0.54–2.72), progression of spondylolisthesis (HR 0.59, 95% CI 0.13–2.65), coronal deformity (HR 2.18, 95% CI 0.48–10.01), central stenosis (HR 1.35, 95% CI 0.21–8.61), composite degeneration (HR 1.76, 95% CI 0.87–3.56), or reoperation (HR 0.44, 95% CI 0.12–1.62). Exploratory subgroup analysis suggested that minor (grade 1) violations may contribute to axial instability, whereas full joint traversal (grade 2) may confer relative stability, though neither reached statistical significance.

**Conclusions:**

In this cohort with extended follow-up, superior FJV was not significantly associated with any measure of radiographic degeneration or reoperation. These findings suggest that FJV may not be a major determinant of long-term outcomes after lumbar fusion. Further biomechanical and clinical studies are warranted to clarify whether specific grades of FJV differentially affect cranial segment stability and screw performance.

**140 characters:**

Superior FJV on CT didn’t significantly increase hazards of disc height loss, listhesis, stenosis, deformity, or reop after fusion.

## Introduction

Pedicle screw fixation has become the gold standard for achieving segmental stability when treating unstable lumbar pathology. By engaging all 3 spinal columns, pedicle screws improve fusion rates compared to earlier methods of internal fixation [1]. However, accurate screw placement remains technically demanding, particularly with minimally invasive techniques where direct anatomical visualization is limited [2].

A well-recognized complication of pedicle screw fixation is superior facet joint violation (FJV), which occurs when a screw breaches the boundaries of 1 or both facet joints above the construct. FJV is frequently encountered in clinical practice and has been implicated as a potential driver of adjacent segment disease (ASD), the most common long-term complication after lumbar fusion [3]. ASD occurs in up to 4% of patients annually [4], yet its pathogenesis is incompletely understood, with proposed mechanisms including increased loading across adjacent facets and discs after fusion, postoperative changes in sagittal balance or simply progression of the underlying degenerative process [5]. It is believed FJV may also contribute to ASD by altering the biomechanics of the cranial segment [6]. One common argument in favor of navigated and robotic-assisted screw placement is the ability to prevent FJV, with the expectation that this may translate into lower rates of ASD [7,8].

Experimental studies suggest that FJV can modify range of motion and load transmission in the superior adjacent segment, but findings are inconsistent and may depend on the extent of cortical or articular surface involvement [3]. Clinical studies similarly report heterogeneous results: some identify FJV as an independent predictor of radiographic ASD or reoperation, while others find no significant association. Limitations of the existing evidence include heterogeneous definitions of FJV, inadequate adjustment for confounders, selection bias, poor quality analyses and a lack of replication [3].

Accurate grading of screw position requires postoperative CT, which is typically only performed when there is clinical concern. Prior studies [9] limited to patients with postoperative CT spine are therefore prone to selection bias, due to a high prevalence of symptoms and complications. In contrast, this retrospective cohort includes both incidental and dedicated postoperative imaging of the lumbar spine, reducing this bias and providing a more representative sample of the postfusion cohort. The study aims to quantify the incidence of radiological degeneration at the superior segment following pedicle screw fixation and to determine whether superior FJV is associated with increased risk of disc height loss, coronal deformity, spondylolisthesis, canal stenosis, or reoperation

## Methods

### Study design and participants

This retrospective cohort study was performed with institutional review board approval from a tertiary referral centre (1/378/2359) and is reported according to the Strengthening the Reporting of Observational Studies in Epidemiology (STROBE) guidelines [10].

Patients admitted between 1st January 2010 and 1st January 2025 who underwent lumbar pedicle screw placement for degenerative or traumatic indications, and who subsequently had a CT scan capturing the instrumented lumbar levels, were eligible for inclusion. Exclusion criteria were malignant or infectious spine pathology and inadequate scan quality or coverage. All patient data were anonymized at source, and imaging assessments were stored in a secure database.

### Imaging acquisition, radiological assessments and endpoints

**Fig. 1:**
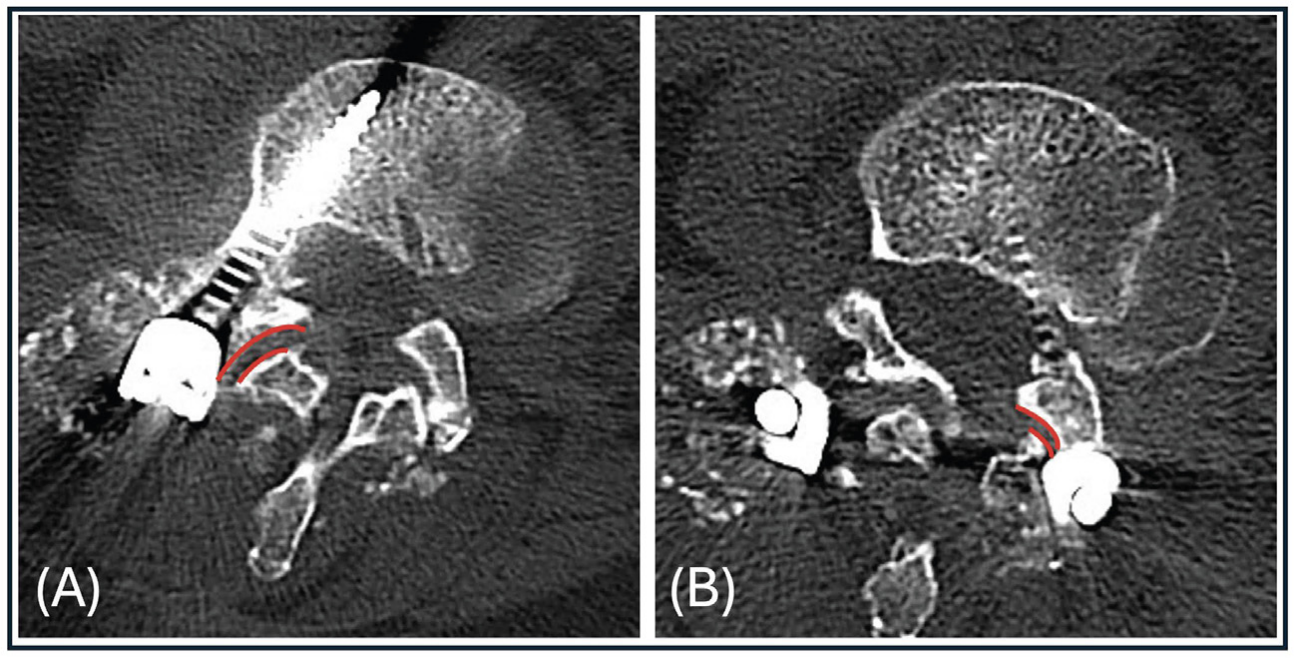
Example grade 1 and grade 2 FJV.

All CT imaging was performed using 64-detector MDCT scanners with patients supine. Digital sagittal and coronal reconstructions were available for all studies. FJV was graded according to the classification described by McNamee et al. [3]: (1) Grade 0 = no violation. (2) Grade 1 = screw breaching the lateral cortex of the superior facet without entering the joint space. (3) Grade 2 = screw traversing the joint to involve both articular surfaces. This is a simplification of the system suggested by Babu et al. which has been shown to have good intra- and interrater performance [11]. Example grade 1 and 2 violations are shown in Fig. 1:

Fig. 1A shows right grade 1 FJV with the screw partially penetrating the outer cortex. 1B shows left FJV with the screw traversing both processes.

All scans were reviewed by authors CM and BM who had undergone specific training in the assessment of FJV and radiological measures of degeneration.

Preoperative and last follow up erect lumbar radiographs were assessed for: (1) Intervertebral disc height (IVDH): measured at the disc midpoint on lateral radiographs as the vertical distance between adjacent endplates, (2) Spondylolisthesis: Calculated as the ratio of antero-/retrolisthesis of the superior vertebra relative to the anteroposterior diameter of the lower vertebral body at its midpoint, (3) Coronal deformity: Quantified by Cobb angles between the most angulated vertebrae above and below the principal curve, where present

At final follow up CT, additional superior segment parameters were measured, including the anteroposterior diameter of the canal at the infra-pedicular margin of the superior vertebra and the anteroposterior diameter of the lateral recesses [12].

The following endpoints were assessed during survival analysis: (1) 20% (≥2mm) reduction in intervertebral disc (IVD) height at the superior segment. (2) AP canal diameter <10mm. (3) ≥5% absolute increase in superior segment spondylolisthesis. (4) Increase in Cobb angle ≥5°. (5) A composite of all radiological measures. (6) Reoperation, defined as repeat lumbar decompression, revision of instrumentation or extension of fusion.

### Statistical analysis

Propensity scores, defined as the probability of superior FJV, were estimated using multivariable logistic regression models implemented in PyMC and Python 3. The model incorporated baseline covariates known or suspected to influence FJV and ASD, including age, sex, surgical indication, pelvic incidence, pre- and postoperative sagittal parameters (pelvic tilt, lumbar lordosis, sacral slope), use of an interbody cage, percutaneous vs open screw placement, number of instrumented levels and the use of intraoperative navigation. Inverse probability of treatment weighting (IPTW) was applied to adjust for the nonrandom allocation of patients into FJV and no FJV groups. Propensity scores derived from logistic regression were used to generate a weighted pseudo-population in which measured covariates were independent of FJV status. Weights were normalized to average 1.0 across the sample and were truncated at the 1st and 99th percentiles. Covariate balance between weighted cohorts was assessed using standardized mean differences, with SMD <0.1 considered indicative of adequate balance.

Time-to-event analysis was conducted using the Lifelines package. Kaplan-Meier curves were generated to compare endpoint free survival between groups. Survival distributions were compared via Cox proportional hazards regression with robust variance, with 2-tailed p<.05 considered significant. The unweighted populations were also hypothesis tested as part of a sensitivity analysis. In tables, binary data are summarized as number and percentage, while continuous data are summarized as mean and standard deviation. For univariate comparison of outcomes within tables, the Kruskal-Wallis test was used for multiple group means, while the χ² test was used for binary outcomes. In Figs., displaying Kaplan-Meier curves, the FJV group is shown in pink and the no violation group is shown in blue.

## Results

Over the review period, 1,000 lumbar fusions were performed, of which 74 patients underwent dedicated or incidental CT imaging that included the lumbar spine. Three were excluded due to neoplastic or infectious pathology, leaving 71 patients with postoperative CT scans capturing both the pedicle screw construct and the superior adjacent segment for analysis. No patient underwent laminectomy at the cranial level prior to imaging assessment.

FJV at the superior segment was identified in 35 patients. Among these, 21 patients (29.6%) had unilateral grade 1 violation without grade 2 involvement, while 14 patients (19.7%) had unilateral or bilateral grade 2 violation. Median event free follow up was 3.9 years (IQR 2.17 – 7.61 years) with a maximum of 15.2 years and a minimum of 2.6 weeks. The upper instrumented level was T12 in 1 patient (1.4%), L1 in 4 (5.6%), L2 in 15 (21.1%), L3 in 17 (23.9%), L4 in 29 (40.8%), and L5 in 5 (7.0%). 38 fusions were single level (53.5%), 19 were 2 levels (26.8%), 12 included 3 levels (16.9%) and 2 involved 4 levels (2.8%). After IPTW, the effective sample size was 45.6 patients. Adjusted and weighted baseline characteristics are presented in Table 1.

**Table 1:** Baseline characteristics before and after inverse probability of treatment weighting

		Before IPTW				After IPTW		
		No FJV (*N*=36)	Superior FJV (*N*=35)	SMD	p	No FJV (Weighted *N*=86.3)	Superior FJV (Weighted *N*=83.3)	SMD
Age at time of surgery		64.9±15.3	66.0±12.4	0.080	.743	67.6±15.0	67.3±10.6	0.028
Male sex		14 (38.9%)	16 (45.7%)	*0.138*	.561	31.8 (36.8%)	36.1 (43.3%)	*0.133*
Degenerative pathology		22 (61.1%)	27 (77.1%)	*0.352*	.144	62.4 (72.3%)	62.4 (74.9%)	0.060
Intraoperative navigation		9 (25.0%)	19 (54.3%)	*0.591*	**.019**	30.1 (34.9%)	32.9 (39.5%)	0.094
Interbody cage		3 (8.33%)	9 (25.7%)	*0.463*	.081	18.0 (20.8%)	18.5 (22.2%)	0.033
Number of instrumented vertebrae		2.92±0.94	2.31±0.87	*0.677*	**.006**	2.66±0.85	2.74±0.99	0.085
Percutaneous screw placement		1 (2.78%)	4 (11.4%)	*0.347*	.154	2.42 (2.80%)	5.58 (6.70%)	*0.186*
Pelvic Incidence		55.9±10.0	57.1±13.4	*0.107*	.669	57.4±9.69	57.7±12.3	0.027
LL	Preoperative	40.7±11.5	41.9±13.6	0.097	.669	43.6±10.9	41.2±12.2	0.207
	Postoperative	41.1±10.9	43.9±14.7	*0.226*	.358	43.6±10.1	44.3±14.3	0.058
SS	Preoperative	33.8±8.16	32.5±7.14	*0.179*	.479	34.6±7.89	33.6±7.01	*0.133*
	Postoperative	32.9±7.94	31.9±8.40	*0.131*	.592	33.2±7.16	33.8±8.52	0.080
PT	Preoperative	25.0±7.41	23.6±9.87	*0.168*	.523	24.6±7.24	24.3±10.2	0.030
	Postoperative	23.8±8.11	25.4±10.5	*0.174*	.488	25.5±8.52	24.5±10.6	*0.106*

Covariates with significant differences between groups shown in bold, standardized mean difference (SMD) >0.1 are italicized. LL, lumbar lordosis; SS, sacral slope; PT, pelvic tilt.

Prior to weighting, 2 covariates, use of intraoperative navigation and the number of instrumented vertebrae, differed significantly between groups, and 12 covariates demonstrated imbalance with SMD >0.1. After IPTW, covariate balance improved across all quantities except preoperative LL. Residual imbalance (SMD >0.1) was observed only for male sex, percutaneous screw placement, preoperative LL and postoperative PT, while all other covariates were adequately balanced.

### Univariate analysis

Univariate hypothesis testing of the unweighted groups revealed no significant differences between FJV grades in intervertebral disc height loss, progression of spondylolisthesis, change in coronal alignment, AP canal diameter, lateral recess height or reoperation rate. This is shown in Table 2.

**Table 2:** Univariate analysis of final radiographic outcomes by superior FJV grade

	Grade 0	Grade 1	Grade 2	p
IVD height loss (mm)	2.18±2.73	1.71±2.44	0.53±1.11	.111
Delta vertebral slip (%)	1.96±5.18	0.18±4.51	1.46±4.78	.422
Delta cobb angle (°)	0.65±6.49	2.96±5.46	−0.611±4.02	.085
AP canal diameter (mm)	14.3±2.97	14.18±2.77	14.9±3.99	.707
Left lateral recess height (mm)	7.28±2.36	6.60±1.78	6.35±1.74	.512
Right lateral recess height (mm)	7.33±2.32	6.45±1.86	7.62±1.97	.215
Reoperation	10 (27.8%)	3 (14.3%)	2 (14.3%)	.379

Grade 1 includes unilateral grade 1 FJV without contralateral violation, or bilateral grade 1 FJV. Grade 2 includes any uni- or bilateral grade 2 FJV.

### Loss of intervertebral disc height >20% (at least 2mm)

At final follow up, 29 patients (40.8%) demonstrated ≥20% (at least 2 mm) disc height loss at the superior segment compared with baseline radiographs. This included 17 events in the no-FJV group and 12 in the FJV group. After IPTW, event counts corresponded to 40.6 in the no-FJV group and 35.5 in the superior FJV group.

**Fig. 2:**
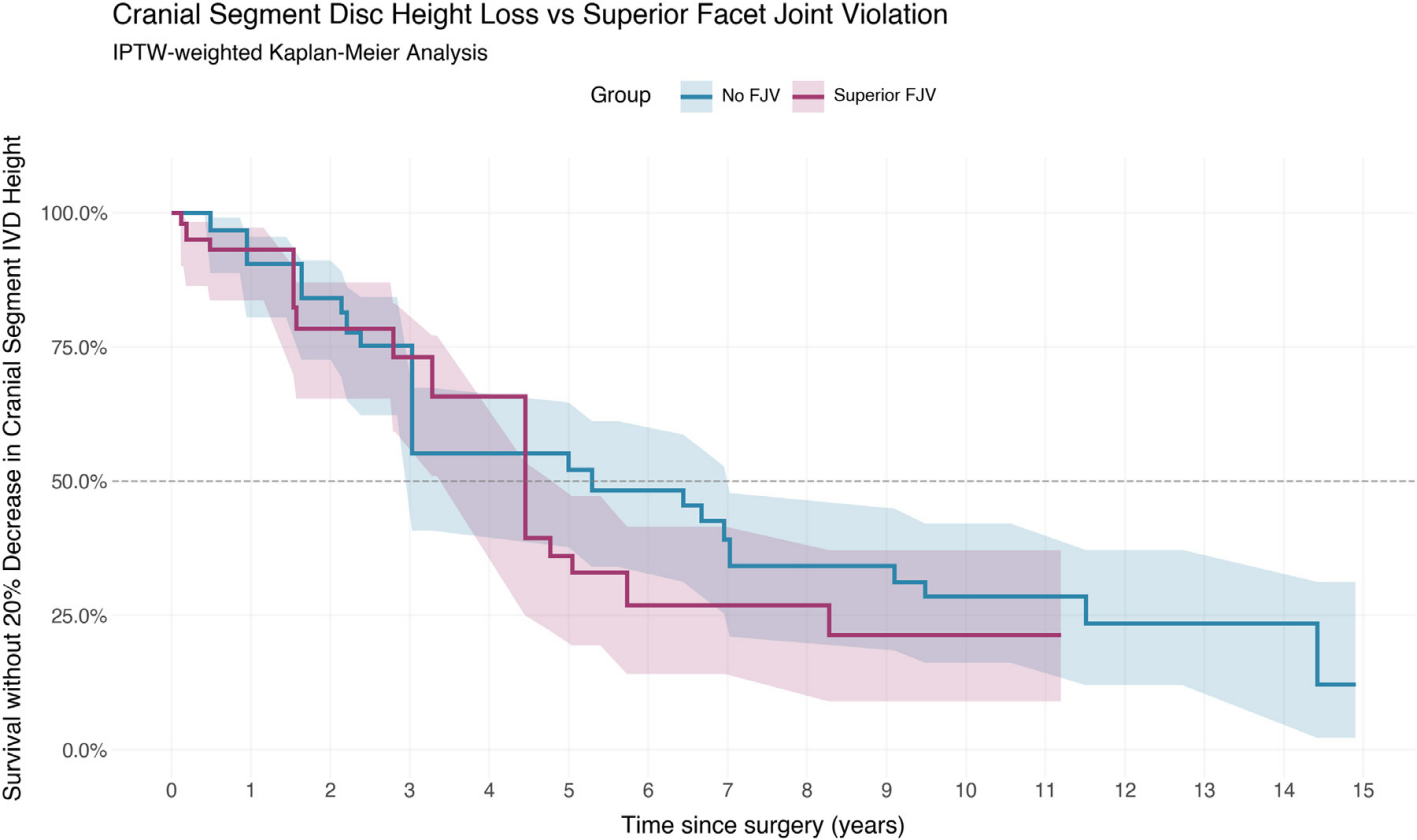
Superior segment disc height loss vs superior facet joint violation.

Weighted Cox proportional hazards model with robust variance showed no significant association between FJV and disc height loss (HR 1.21, 95%CI 0.54 – 2.72, p=.65). Similarly, unweighted analysis showed no significant difference (HR 1.35, 95%CI 0.62 – 2.97, p=.45). This is displayed in Fig. 2.

### Spondylolisthesis of superior segment

At final follow-up, 10 patients (14.1%) showed a 5% or greater absolute increase in vertebral slip at the superior segment. 6 events occurred in the no-FJV group and 4 events in the FJV group. After weighting, there were 19.3 events in the no-FJV group and 8.5 in the FJV group.

**Fig. 3:**
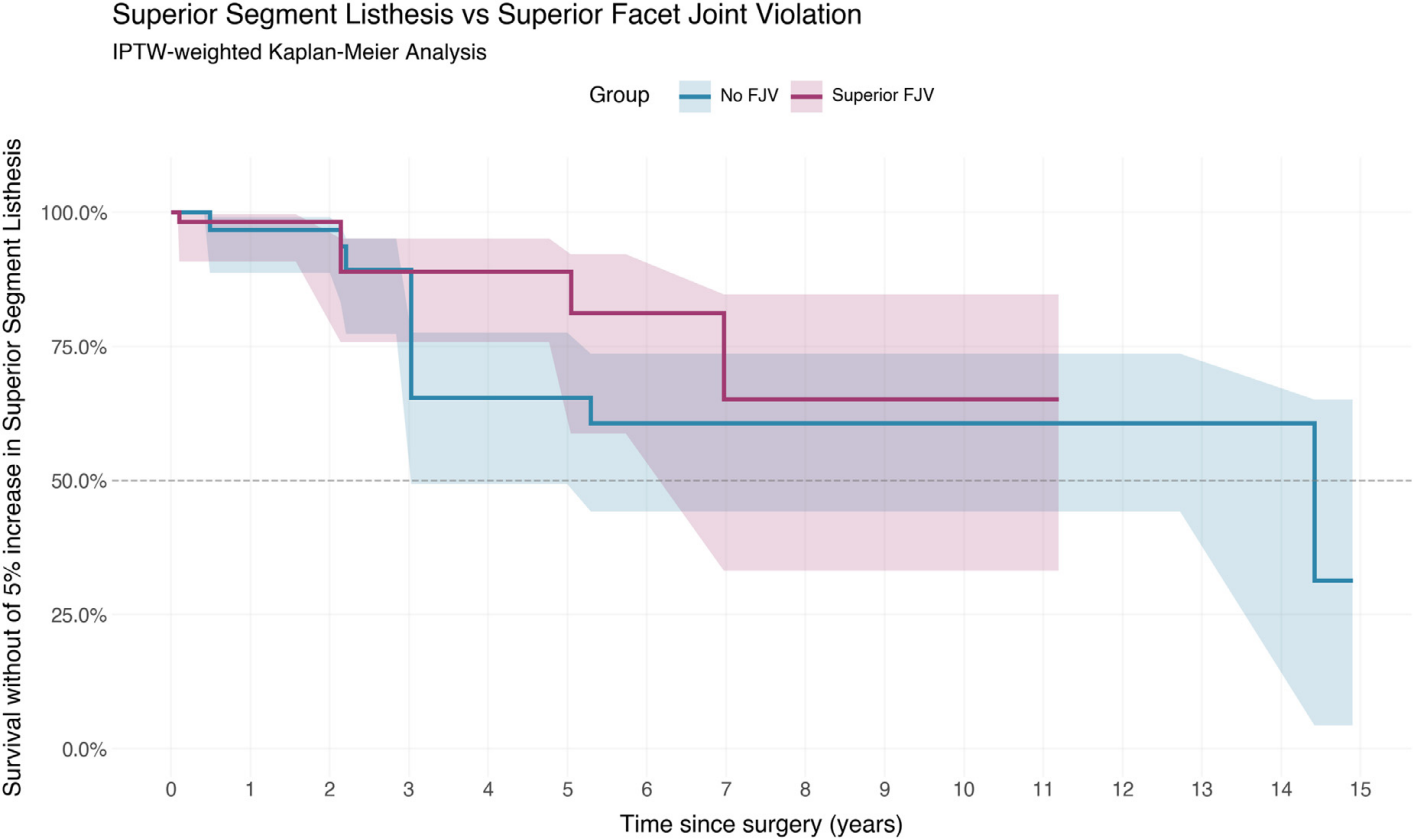
Superior segment spondylolisthesis vs superior facet joint violation.

On weighted analysis, the Cox proportional hazards model showed no significant association between FJV and progression of listhesis (HR 0.59, 95% CI 0.13 – 2.65, p=.49). Interpretations from unweighted regression were similar (HR 1.15, 95% CI 0.30–4.38, p=.84). This is shown in Fig. 3.

### Onset/progression of coronal deformity

At final follow up, 14 patients (19.7%) developed a new or progressive coronal deformity, defined as an increase in Cobb angle of ≥5°. There were 6 events in the no-FJV group and 8 in the FJV group, corresponding to 19.9 and 23.8 weighted events respectively.

**Fig. 4:**
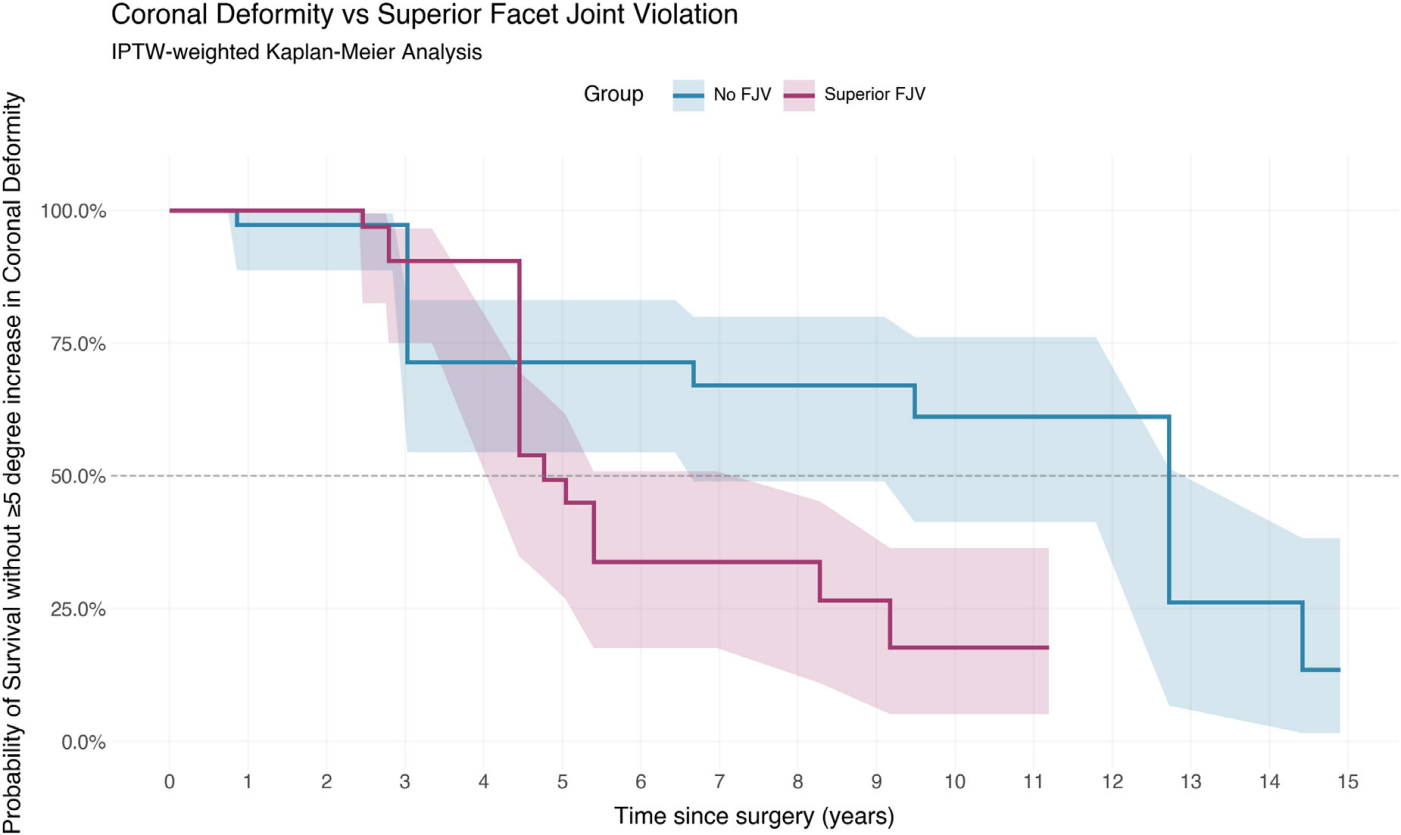
Coronal deformity vs superior facet joint violation.

Weighted Cox regression showed no significant association between FJV and the development of coronal deformity (HR 2.18, 95% CI 0.48- 10.01, p=.32). In contrast, unweighted analysis suggested a significant association (HR 3.72, 95%CI 1.06-12.98, p=.04). This is illustrated in Fig. 4.

### Central stenosis of the superior segment

At last follow up, 5 patients (7.0%) had an AP canal diameter ≤10 mm at the superior segment. This included 2 events in the no-FJV group and 3 in the FJV group. After weighting, this corresponded to 3.5 and 4.6 events in the no-FJV and FJV groups respectively.

**Fig. 5:**
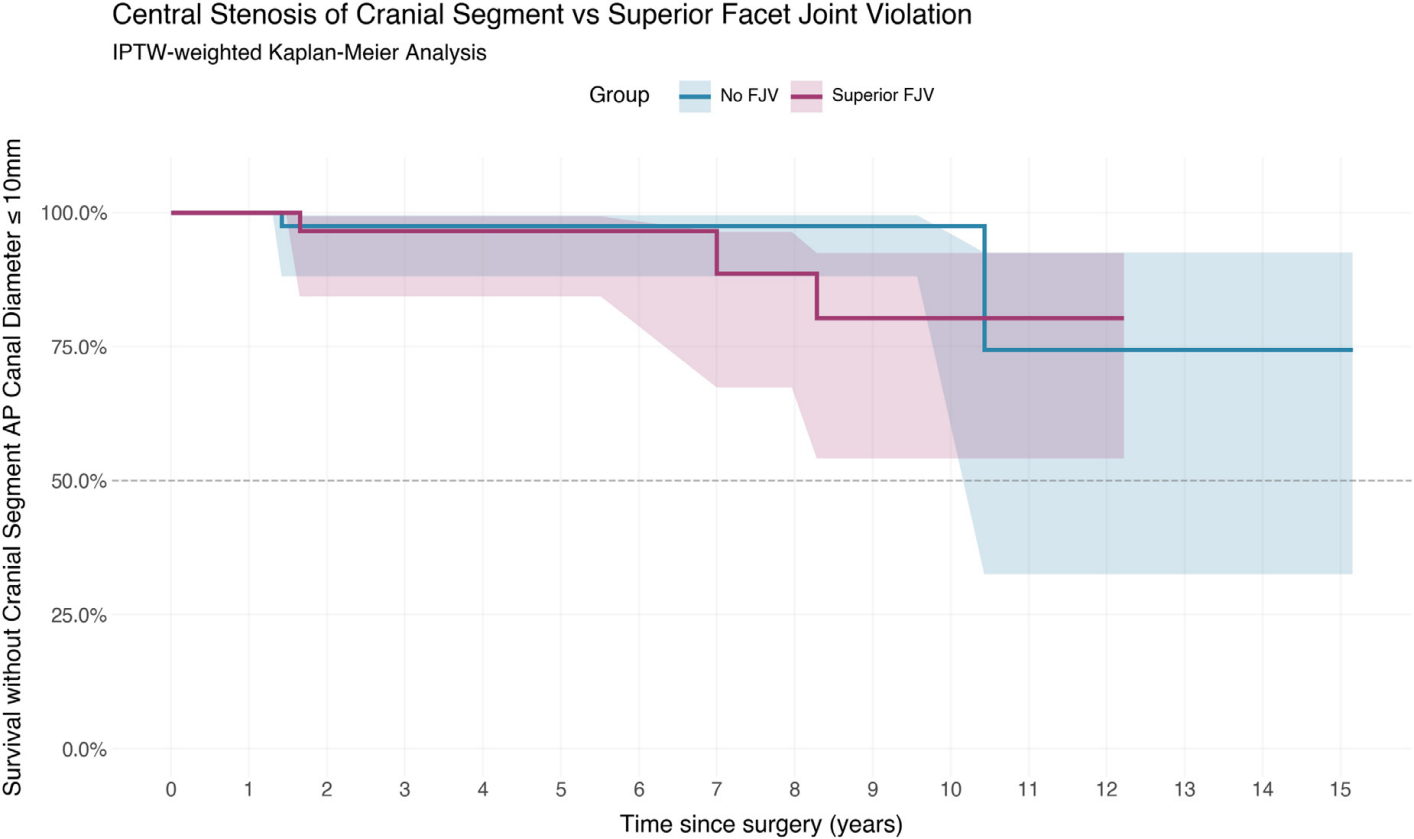
Central stenosis of the cranial segment vs superior facet joint violation.

Weighted Cox regression with robust variance showed no significant relationship between FJV and canal narrowing (HR 1.35, 95% CI 0.21–8.61, p=.58). Unweighted analysis also failed to demonstrate a significant difference (HR 4.77, 95% CI 0.52–43.96, p=.17). These findings are shown in Fig. 5.

### Composite endpoint: radiographic degeneration

Across all markers of degeneration, 39 patients experienced at least 1 event at the superior segment. This included 20 raw events in the no-FJV group and 19 in the FJV group. After weighting, events equaled 48.5 in the no-FJV group and 50.7 in the FJV group.

**Fig. 6:**
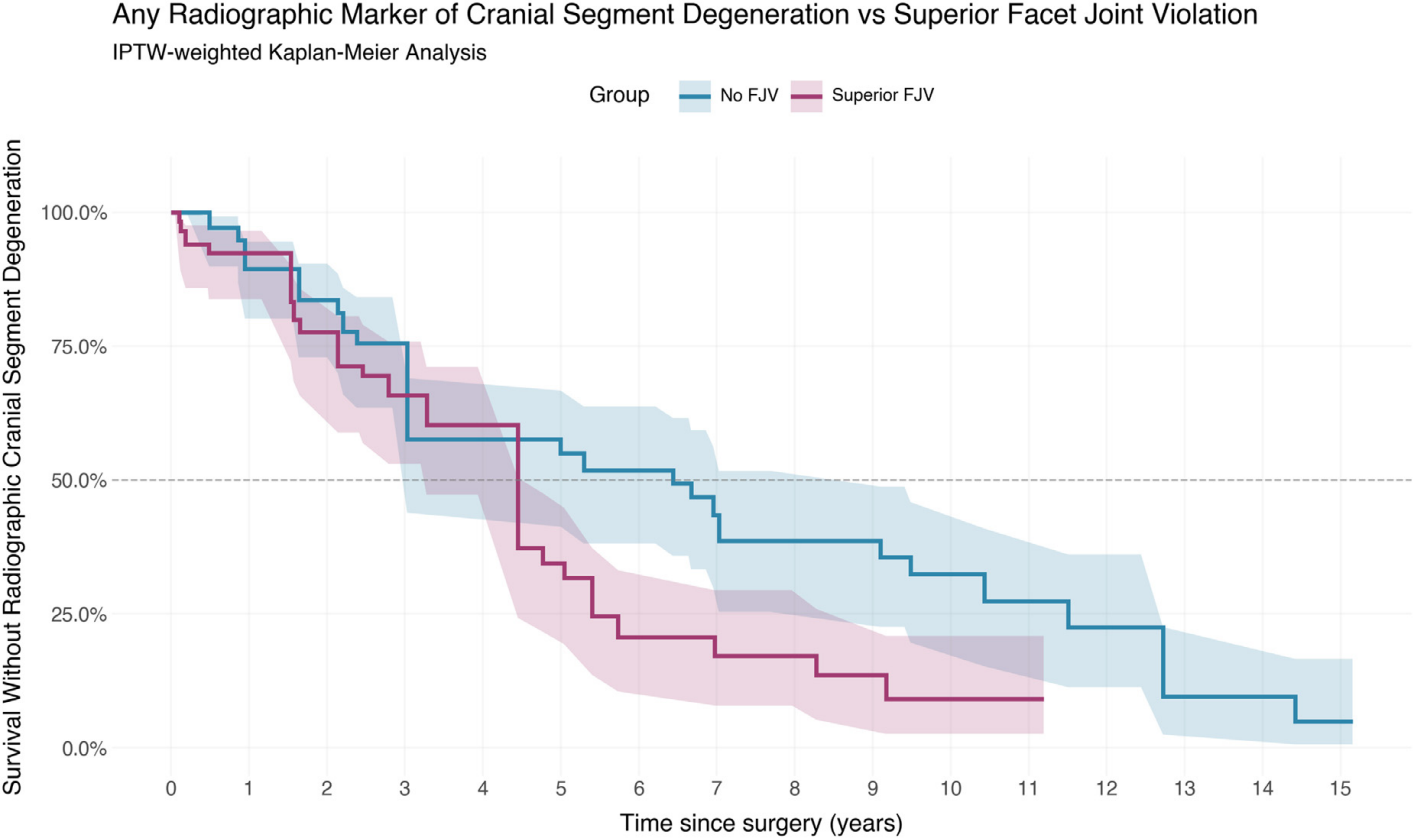
Composite radiographic degeneration vs superior facet joint violation.

On weighted analysis, the Cox proportional hazard model did not show a statistically significant association between FJV and composite degeneration (HR 1.76, 95% CI, 0.87-3.56, p=.12). Unweighted analysis suggested a stronger relationship (HR 2.15, 95% CI, 1.07-4.34, p=.03) reaching significance. This is shown in Fig. 6.

### Revision surgery

During the follow-up period, 15 patients (21.1%) underwent reoperation at the index or superior segment. Of these, 12 procedures were repeat decompressions with or without extension of fusion for adjacent segment disease, 2 were for revision of loose metalwork (of which 1 patient had superior FJV while the other did not), and 1 was performed for correction of new degenerative scoliosis.

**Fig. 7:**
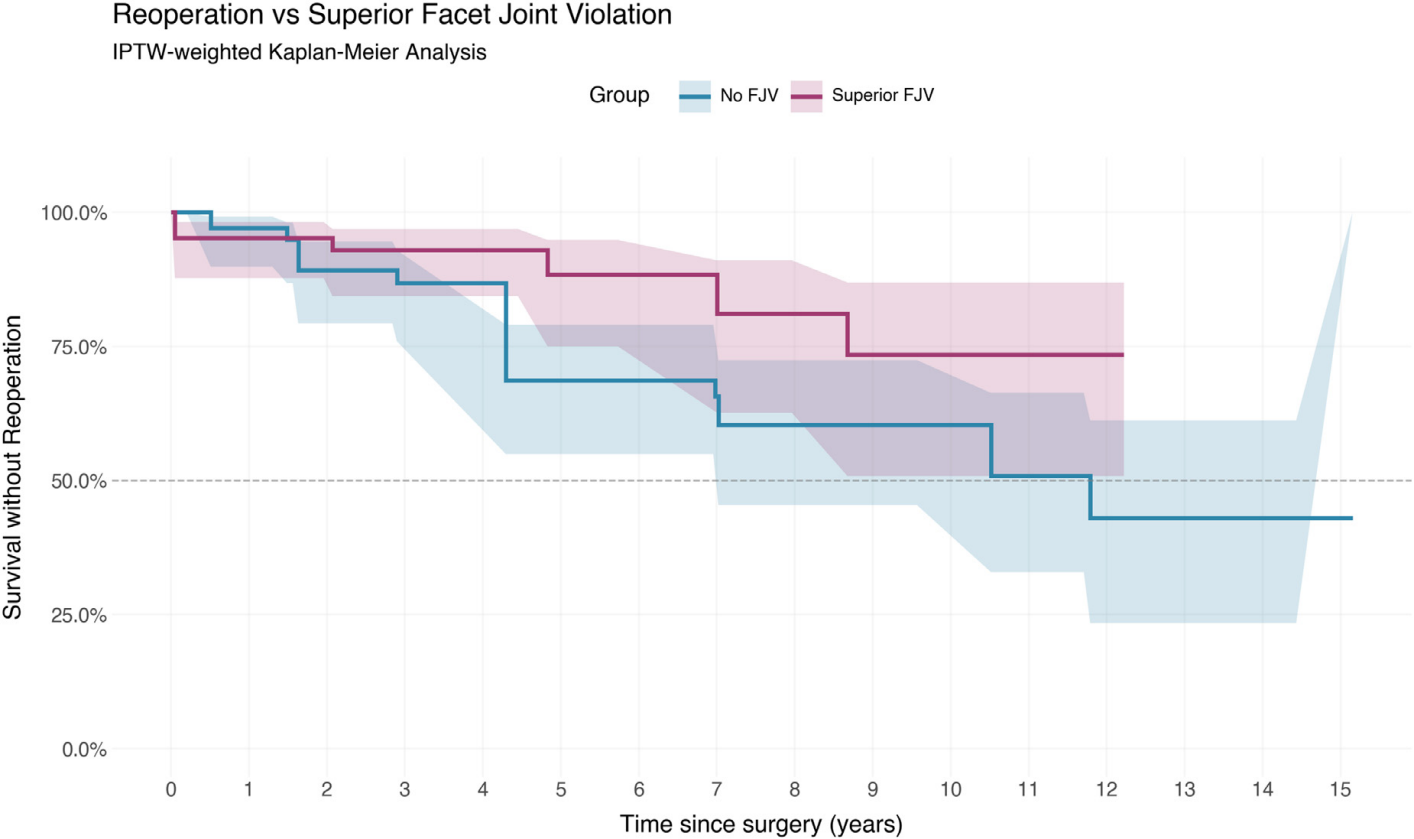
Revision surgery vs superior facet joint violation.

There were 10 revision events in the no-FJV group and 5 in the FJV group, corresponding after weighting to 27.3 and 10.0 events respectively. On Cox regression, there was no significant difference in revision risk. (HR 0.44, 95% CI 0.12 −1.62, p=.22). Unweighted analysis also did not reach significance (HR 0.81, 95% CI 0.26-2.51, p=.71). This is shown in Fig. 7.

## Discussion

Superior FJV has attracted increasing attention due to its potential role in the development of ASD and because modern technologies such as navigation and robotic assisted screw placement may reduce its incidence [3]. It is therefore important to evaluate the widely held assumption that FJV increases loading at the cranial segment, alters motion and accelerates degeneration.

A recent scoping review highlights the limitations of current evidence [3]. Biomechanical studies have produced conflicting results, with some suggesting that FJV restricts motion and others indicating that it increases mobility. It is frequently cited that FJV raised intra-discal pressure at the superior segment, however this finding is derived from a single finite element model which remains unvalidated in animal or human studies. Clinical investigations are similarly inconsistent. Of 6 studies specifically investigating this phenomenon, 5 reported significant associations between FJV and ASD, but 2 contained calculation or reporting errors and another was at high risk of selection bias. Only 1 published study adjusted for sagittal alignment as part of a multivariate analysis [3].

This study aimed to address these limitations. Patients with incidental lumbar spine CT imaging obtained for nonspine reasons were included, thereby reducing the selection bias associated with patients who receive dedicated spine imaging [13]. Because pre-existing facet arthropathy may both predispose to violation and accelerate superior segment degeneration, IPTW was employed to balance degenerative and traumatic cases, along with other measured confounders between the FJV and no-FJV groups. Furthermore, because ASD is a time dependent process, survival analysis was used to account for the effects of censoring and variable follow up.

We did not observe a statistically significant association between FJV and subsequent radiographic degeneration or reoperation after IPTW adjustment; however, confidence intervals were wide for several endpoints, so modest associations cannot be excluded. Across weighted analyses, FJV was not significantly associated with disc height loss, spondylolisthesis, canal narrowing, lateral recess narrowing, coronal deformity, composite degeneration or reoperation. Disc height loss, possibly the most sensitive radiographic marker of degeneration on erect Xray [14,15], showed no difference between groups; in fact the largest mean reduction was observed in patients without FJV. Similarly, no significant association was seen with lateral recess height, despite expectations that violation might predispose to facet joint degeneration. Progression of spondylolisthesis and central stenosis were uncommon in all groups and therefore definitive statements cannot be made.

Coronal deformity and aggregate degeneration showed significant differences in unadjusted analyses that disappeared after weighting, suggesting either confounding in the unadjusted model or lack of power in the weighted cohort. Mean Cobb angle change, stratified comparisons by FJV grade, raise the possibility that grade 1 violations, which breach the capsule but not both articular surfaces, may destabilize the segment, whereas grade 2 violations may stabilize it via the screw’s interaction with both joint surfaces. This effect did not reach significance within this series but remains an interesting possibility that is further alluded to by biomechanical work showing the facet joints and their capsules play a significant role in stabilizing the spine during axial rotation [16].

Weighted composite radiographic degeneration progressed similarly in both groups and may support the view that degeneration is largely time driven and not strongly influenced by FJV. Counterintuitively, revision rates were absolutely lower in the FJV group both in weighted and unweighted comparisons but this again did not reach significance. It is possible, but has not been shown, that FJV increases the screw’s cortical purchase as it traverses the joint and therefore confers greater fixation strength. This is 1 plausible explanation for the reduced revision rate within the FJV group that is deserving of further investigation.

In aggregate, this study fails to find evidence that superior FJV is associated with long term radiographic degeneration or reoperation in the lumbar spine. This finding aligns with 2-year radiographic outcomes reported after a randomized controlled trial comparing robotic assisted versus freehand lumbar fusion [17], but diverges from earlier observational data. The discrepancy may be explained by residual confounding in prior studies, where more degenerated or deformed facets were both more prone to violation and independently linked to worse outcomes. By reducing such bias through weighting, our results suggest the effect of superior FJV, if present, may be smaller than suggested by prior observational studies.

Strengths of this work include extended follow up with survival analysis, the use of causal inference methods to reduce bias inherent to observational studies, and the use of incidental imaging, which likely offers a less biased sample than prior work involving patients who received spinal imaging. The main limitation is sample size, which restricts the power of hypothesis testing, increases the chance of type 2 error and prevents subgroup analysis.

Additional limitations include reliance on CT for assessment of stenosis as this modality poorly visualizes the soft tissues and lacks widely accepted diagnostic criteria for stenosis, however the criteria chosen have been used by other authors for similar purposes [12]. More broadly, there is no universally accepted definition of radiographic ASD; the endpoints and thresholds used here were chosen a priori to reflect commonly described manifestations of degeneration in the lumbar spine and to align with prior work and institutional practice, but other reasonable definitions would likely correlate closely with our measures and thus not materially change the overall findings.

Important covariates such as smoking status, osteoporosis and body mass index were unavailable which likely impart a degree of residual confounding, particularly increased body mass which plausibly predisposes patients to FJV and degenerates the superior segment. Degenerative changes in the superior segment may be level specific, particularly degenerative spondylolisthesis which most commonly affects L4/L5 [18], but stratification was not possible due to small subgroup numbers.

Patients with longer intervals since surgery were also more likely to undergo incidental cross-sectional imaging and therefore to be included in this cohort. We accounted for differences in follow-up using time-to-event methods, and there is no obvious mechanism by which this selection process would differentially influence patients with and without FJV. As such, we expect this bias to have greater impact on generalizability in terms of overall event rates than on the internal comparison between FJV and non-FJV groups, although some residual selection bias cannot be excluded. Radiographic changes do not correlate directly with clinical symptoms but this data was not available.

Heterogeneous scan timing and the absence of systematic follow-up imaging limit inference regarding the temporal sequence of degenerative change. In addition, CT acquisition parameters varied as part of routine care across scanners and protocols; this inter-scan variability may reduce sensitivity to detect modest interval changes, particularly for stenosis-related metrics.

Finally, the study period spans substantial evolution in instrumentation and enabling technologies. Although several technique-related variables (including percutaneous versus open placement, use of intraoperative navigation, interbody use, and number of instrumented levels) were included in the propensity model, unmeasured time-dependent factors could still contribute to residual temporal confounding. Due to sample size, we did not undertake additional stratification or modelling by surgical era, and therefore small technique-related effects on ASD cannot be excluded

In future, finite element models suggesting altered loading after FJV require validation against experimental animal or human cadaveric models and investigators should clarify if grade 1 FJV produces axial instability. Published investigations have shown lateral screw misplacement reduces pullout strength [19]. Similar preclinical and clinical studies are needed to determine whether FJV at any level impacts construct stability through reduced screw loosening or altered fusion rates. The facet capsule contains nociceptors, raising the possibility that FJV contributes to postoperative pain [20]. Levin et al. [9] reported worse PROMs among patients with FJV, although animal studies suggest that capsular strain rather than transection is the main pain generator [20]. The impact of FJV on patient-reported outcomes therefore remains uncertain and merits further investigation.

## Conclusion

In this retrospective cohort study with follow-up extending to 15 years, superior facet joint violation was not significantly associated with radiographic adjacent segment degeneration or reoperation. Although exploratory analyses raised the possibility that minor violations may contribute to axial instability, this effect did not reach statistical significance. Overall, the findings suggest that FJV is unlikely to be a major determinant of long term outcomes after lumbar fusion. Strengths include prolonged follow-up, control of confounding, and the use of incidental imaging, which reduces selection bias. Limitations include small sample size, reliance on CT for stenosis assessment. Future work should validate finite element predictions in cadaveric or animal models, clarify whether minor violations influence stability, and assess the potential impact of FJV on screw loosening, fusion rates and postoperative pain.

## Funding

This work has received funding from the Research Ireland Postgraduate Scholarship Scheme GOIPG/2024/2842.

## Author CRediT Statement

Conceptualisation: C.M., J.M., S.D., J.B. Methodology: C.M., J.M. Formal Analysis: C.M., Resources: S.D, J.B. Data Curation: C.M., B.M. Writing – Original Draft: C.M., B.M. Writing – Review & Editing: R.S., J.M., S.D., J.B. Visualization: C.M.

## Declarations of competing interest

The authors declare that they have no known competing financial interests or personal relationships that could have appeared to influence the work reported in this paper.

## Appendix Supplementary materials

Image, application 1

Image, application 1

Image, application 1

Image, application 1

Image, application 1

Image, application 1

Image, application 1
