# Supplementary figures and images for: Adjacent segment degeneration after superior facet joint violation of the lumbar spine
Source: N Am Spine Soc J. 2025 Dec 22;25:100843. doi: 10.1016/j.xnsj.2025.100843 (PMC12854983; doi:10.1016/j.xnsj.2025.100843)

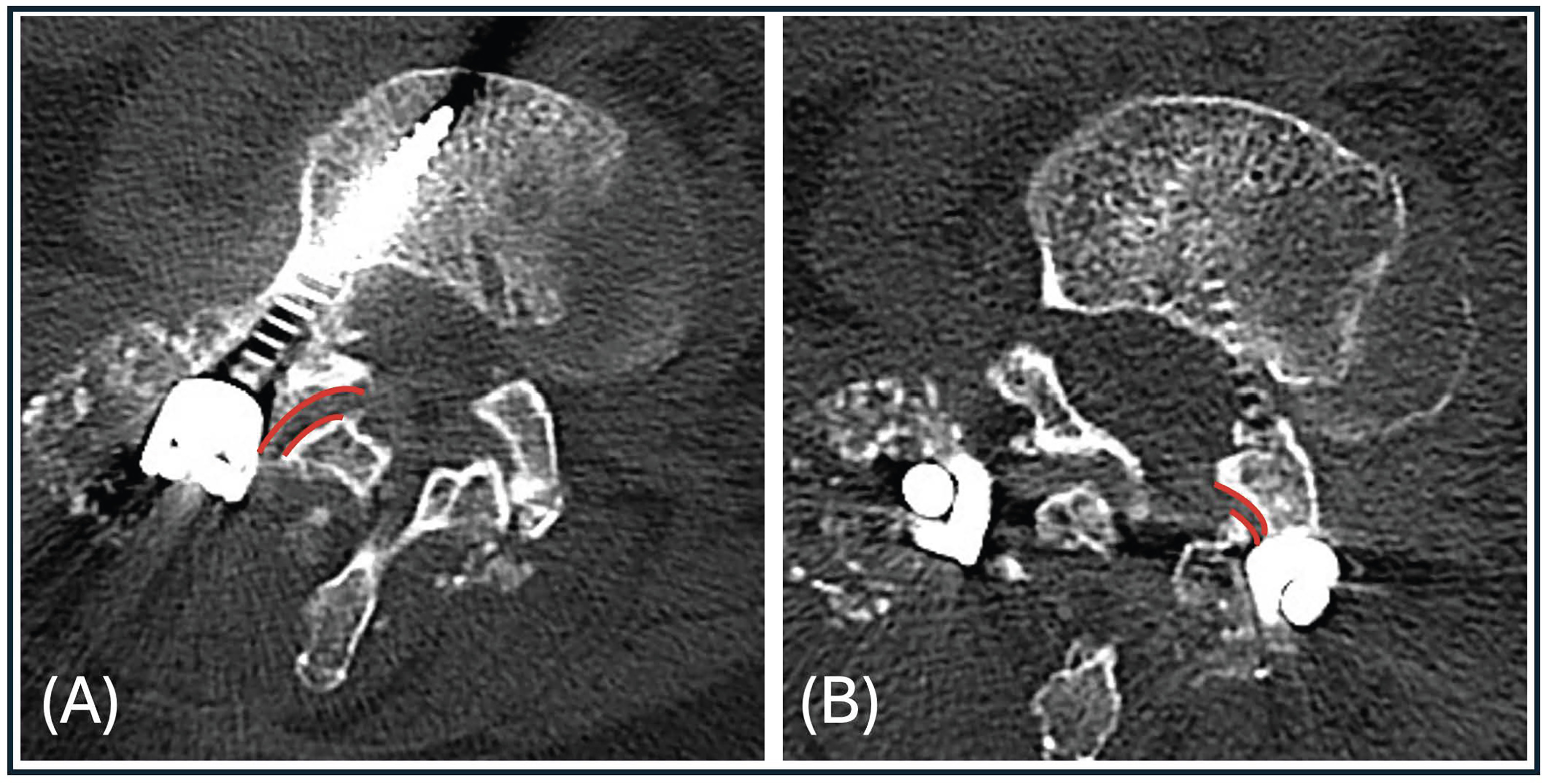

Supplement: Supplementary file 1 [file mmc1.jpg]

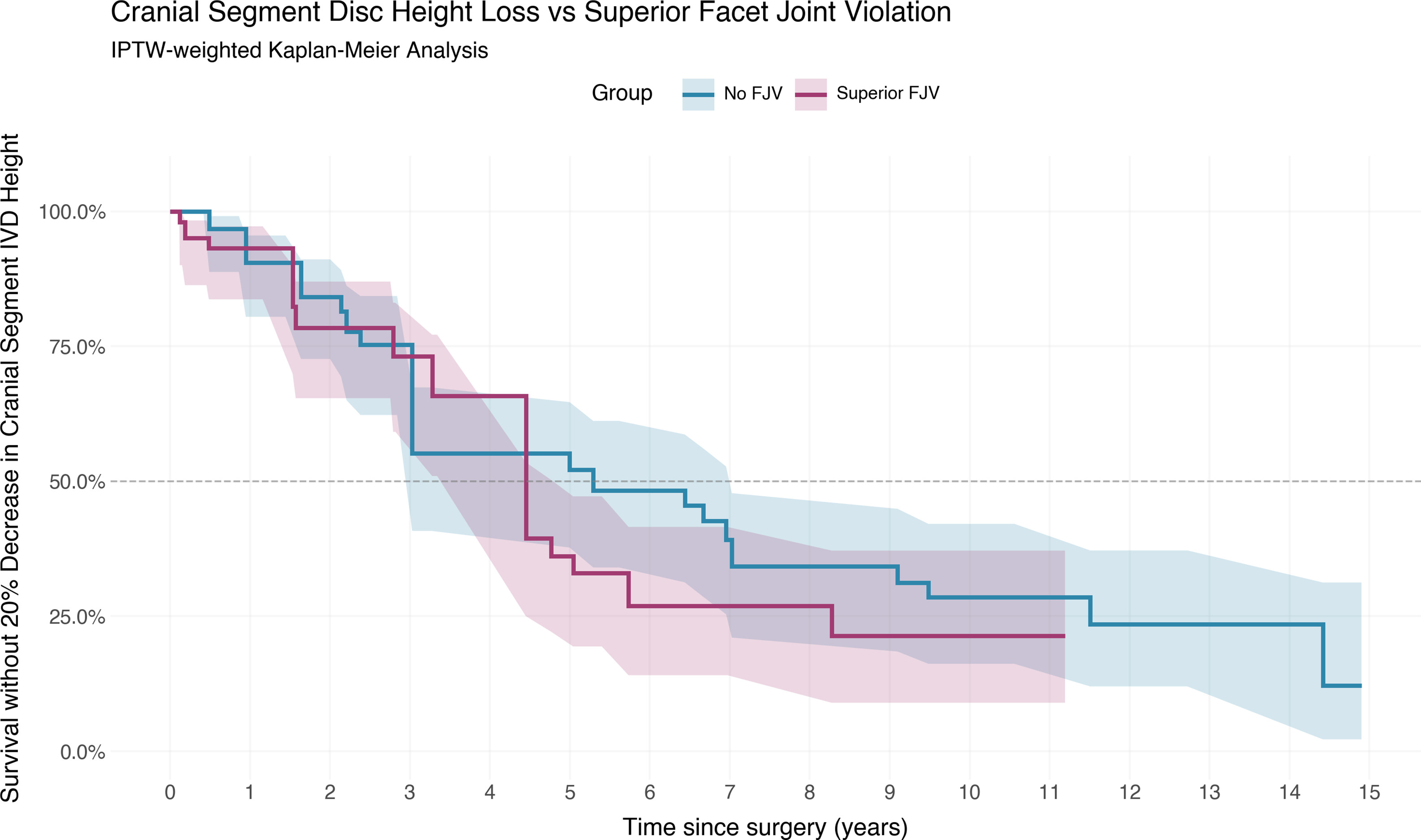

Supplement: Supplementary file 2 [file mmc2.jpg]

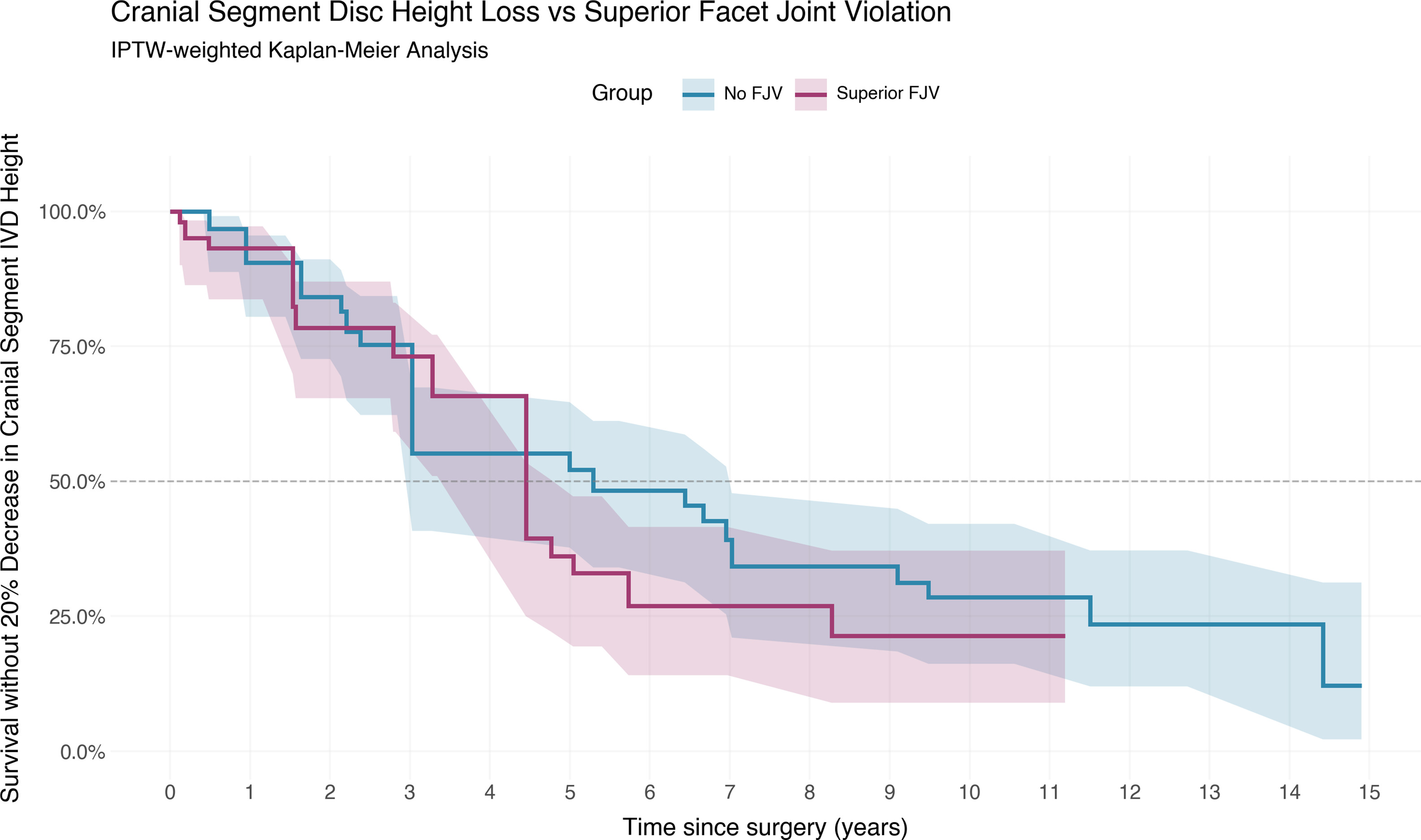

Supplement: Supplementary file 3 [file mmc3.jpg]

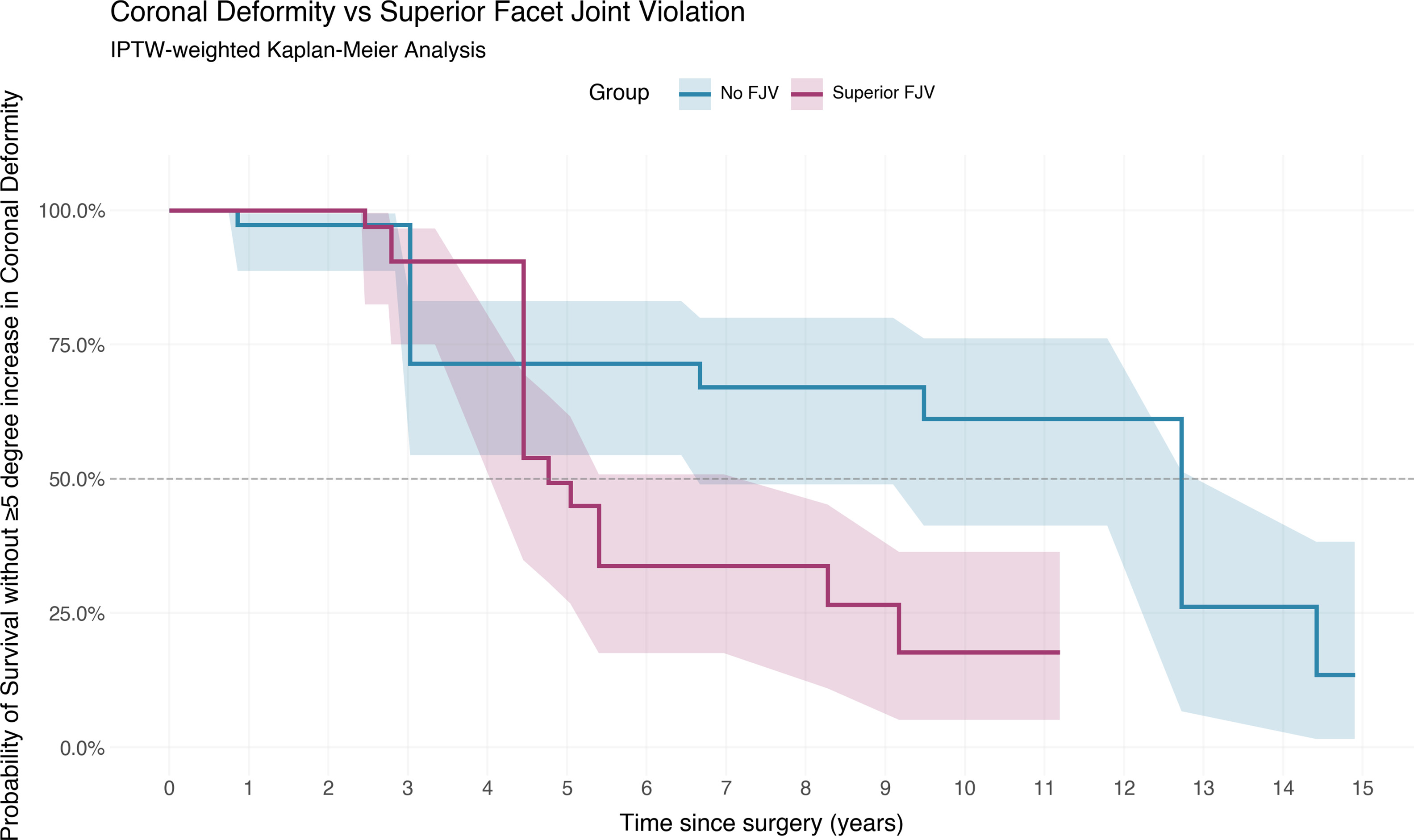

Supplement: Supplementary file 4 [file mmc4.jpg]

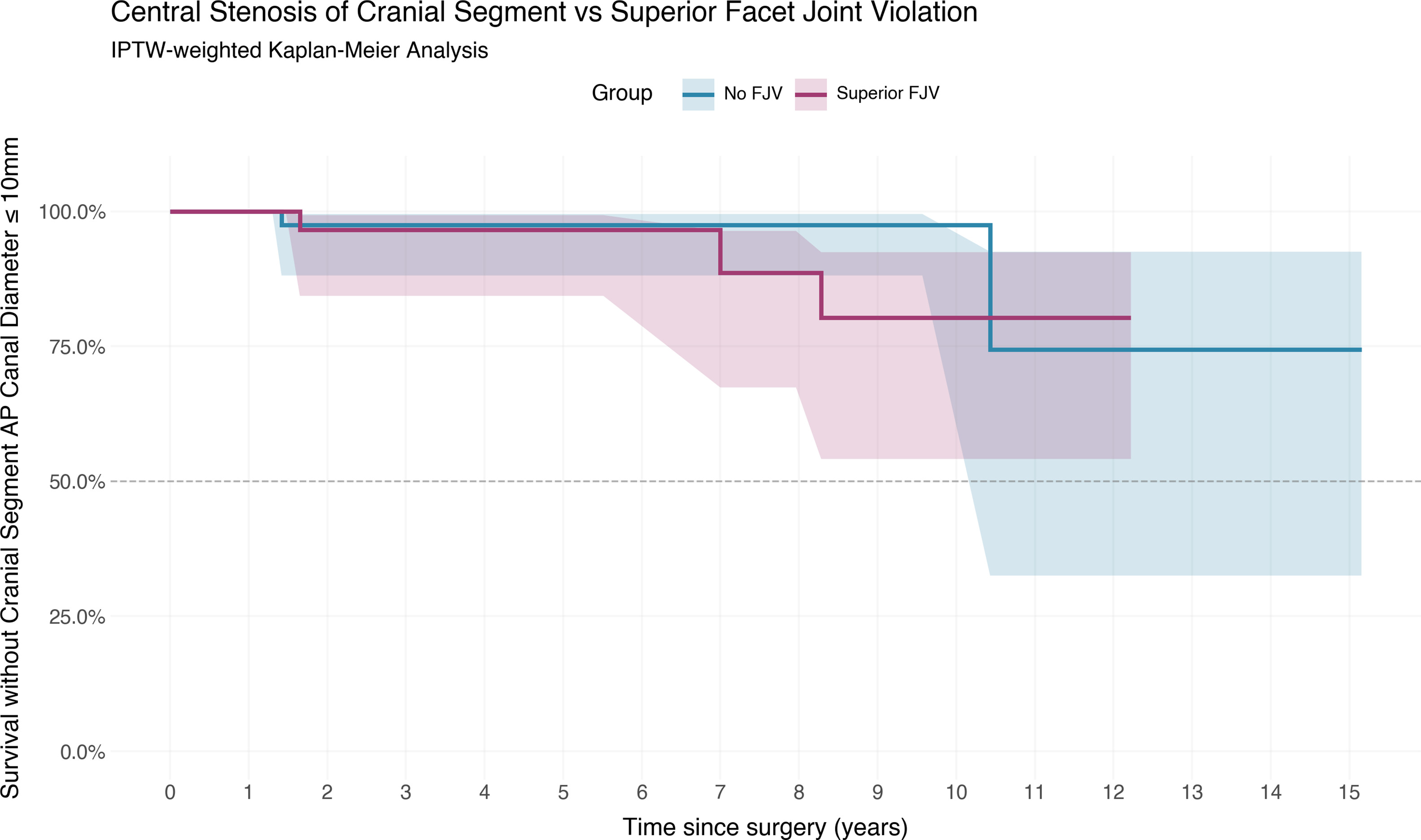

Supplement: Supplementary file 5 [file mmc5.jpg]

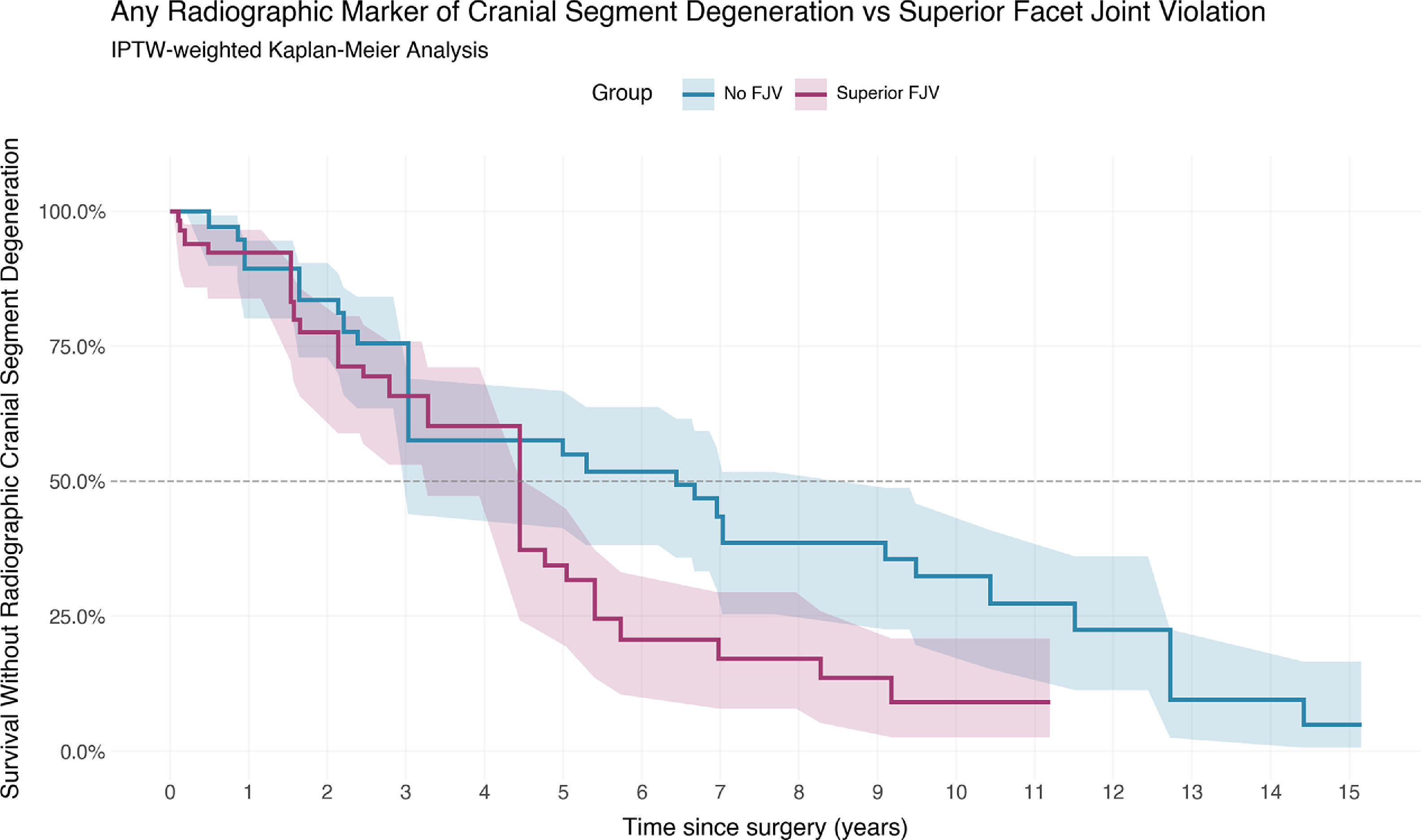

Supplement: Supplementary file 6 [file mmc6.jpg]

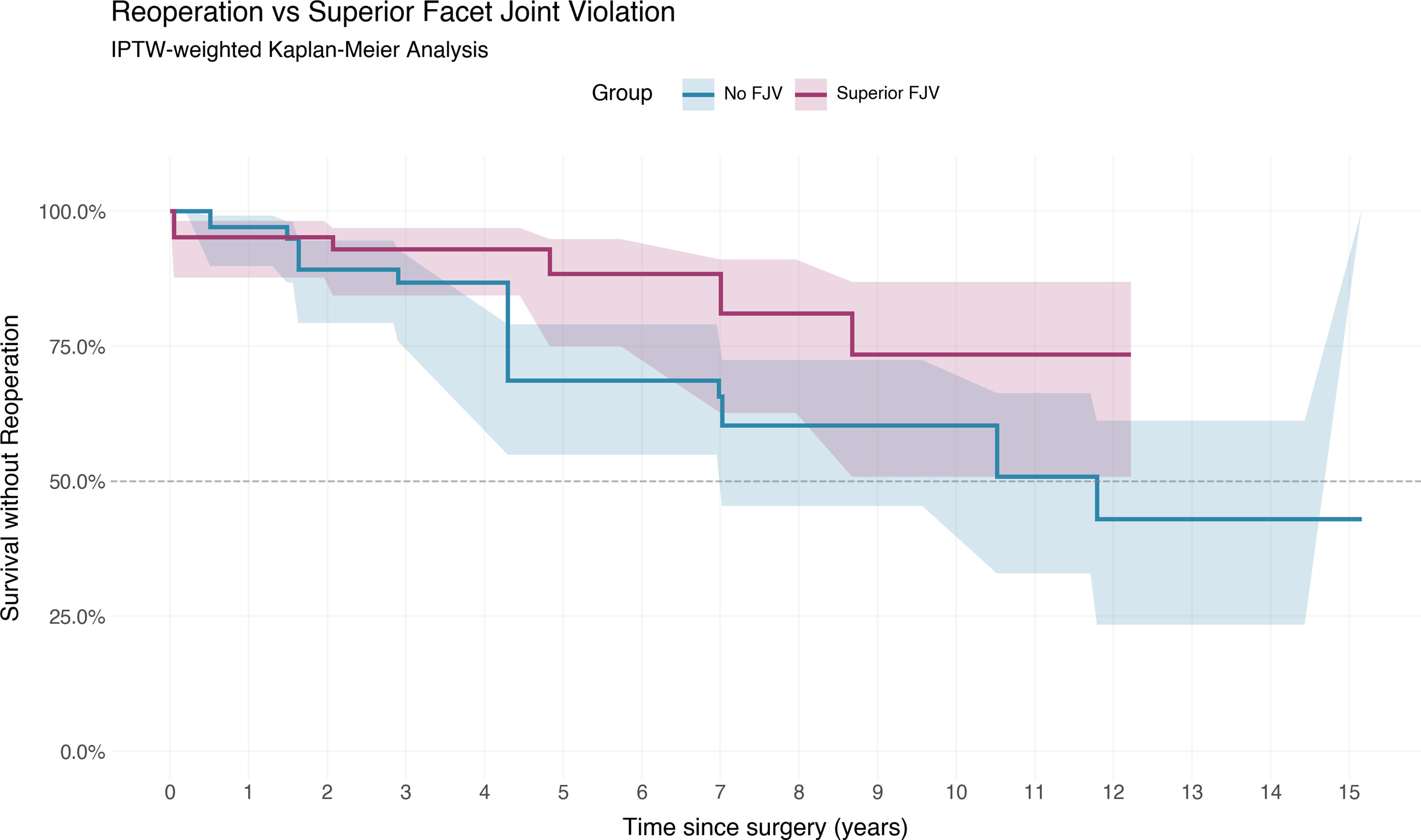

Supplement: Supplementary file 7 [file mmc7.jpg]
